# Structural Features of Heparin and Its Interactions With Cellular Prion Protein Measured by Surface Plasmon Resonance

**DOI:** 10.3389/fmolb.2020.594497

**Published:** 2020-11-26

**Authors:** So Young Kim, Fuming Zhang, David A. Harris, Robert J. Linhardt

**Affiliations:** ^1^Division of Pulmonary and Critical Care, Department of Medicine, University of California, San Diego, San Diego, CA, United States; ^2^VA San Diego Healthcare System, Medical and Research Sections, San Diego, CA, United States; ^3^Department of Chemical and Biological Engineering, Rensselaer Polytechnic Institute, Troy, NY, United States; ^4^Department of Biochemistry, Boston University School of Medicine, Boston, MA, United States; ^5^Department of Chemistry and Chemical Biology, Biological Science and Biomedical Engineering, Center for Biotechnology and Interdisciplinary Studies, Rensselaer Polytechnic Institute, Troy, NY, United States

**Keywords:** heparin, interaction, prion protein, surface plasmon resonance, glycosaminoglycan

## Abstract

Self-propagating form of the prion protein (PrP^*Sc*^) causes many neurodegenerative diseases, such as Creutzfeldt-Jakob disease (CJD) and Gerstmann-Straussler-Scheinker syndrome (GSS). Heparin is a highly sulfated linear glycosaminoglycan (GAG) and is composed of alternating D-glucosamine and L-iduronic acid or D-glucuronic acid sugar residues. The interactions of heparin with various proteins in a domain-specific or charged-dependent manner provide key roles on many physiological and pathological processes. While GAG-PrP interactions had been previously reported, the specific glycan structures that facilitate interactions with different regions of PrP and their binding kinetics have not been systematically investigated. In this study, we performed direct binding surface plasmon resonance (SPR) assay to characterize the kinetics of heparin binding to four recombinant murine PrP constructs including full length (M23–230), a deletion mutant lacking the four histidine-containing octapeptide repeats (M23–230 Δ59–90), the isolated N-terminal domain (M23–109), and the isolated C-terminal domain (M90–230). Additionally, we found the specific structural determinants required for GAG binding to the four PrP constructs with chemically defined derivatives of heparin and other GAGs by an SPR competition assay. Our findings may be instrumental in developing designer GAGs for specific targets within the PrP to fine-tune biological and pathophysiological activities of PrP.

## Introduction

A group of neurodegenerative diseases, including Creutzfeldt-Jakob disease (CJD) and Gerstmann-Straussler-Scheinker syndrome (GSS), are caused by an infectious, self-propagating form of the prion protein, PrP^*Sc*^ (Mercer et al., 2018). PrP^*Sc*^ interacts with a normal, glycophosphatidylinositol (GPI) anchored cellular conformer, PrP^*C*^, on the neuronal surface and induces a conformational change in PrP^*C*^, which leads, through an autocatalytic process, to accumulation of protease-resistant PrP^*Sc*^ in the brain. As part of this process, PrP^*Sc*^ also activates a PrP^*C*^-dependent signal transduction pathway that results in neurotoxicity (Le et al., 2019). This toxic pathway depends critically on the N-terminal domain of PrP^*C*^ (Solomon et al., 2011; Westergard et al., 2011; Wu et al., 2017). Neurotoxicity can be prevented through interactions between N-terminal region of PrP^*C*^ and several ligands, including sulfated glycosaminoglycans (GAGs) and copper ions (Pan et al., 2002; Warner et al., 2002). The PrP^*C*^ molecule is comprised of two major structural domains: a flexible, natively unstructured N-terminal domain (residues 23–127), and a structured, globular C-terminal domain (residues 128–230) (Zahn et al., 2000).

**FIGURE 1:**
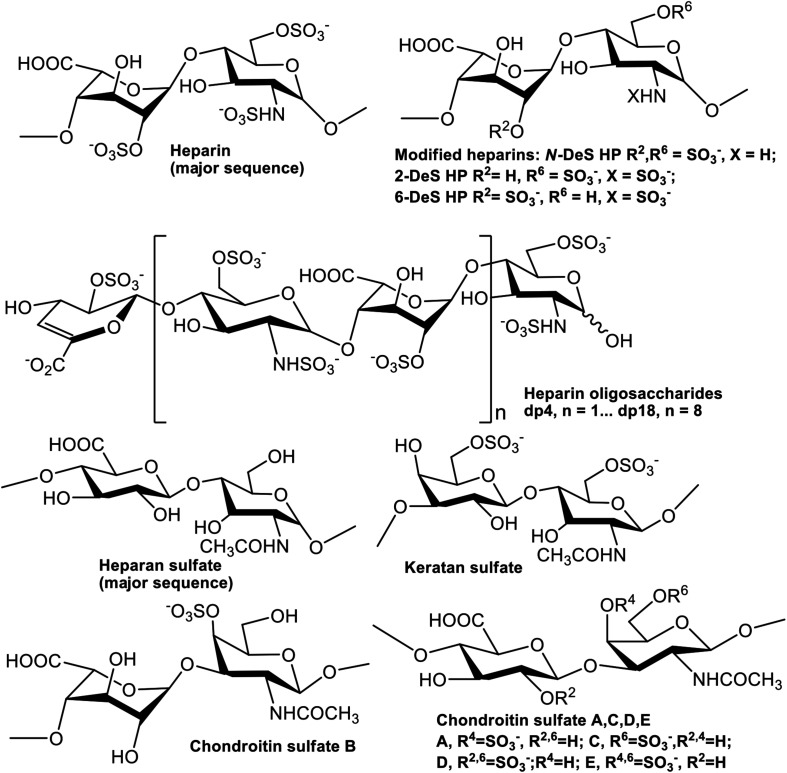
Chemical structures of heparin and heparin-derived oligosaccharides and GAGs.

GAGs are anionic linear polysaccharides comprised of repeating disaccharide units (Figure 1) that are generally found covalently attached to core proteins as proteoglycans (PGs) (Linhardt and Toida, 2004). GAGs in both intracellular and extracellular spaces participate in biological processes such as cellular communication, as well as in the pathogenesis of diseases (Linhardt and Toida, 2004; Kim et al., 2018, 2020). GAG-PrP interactions had been previously reported using human, bovine, and murine PrP (Warner et al., 2002; Andrievskaia et al., 2007; Vieira et al., 2011). Three regions of PrP were identified as sufficient for binding of heparin (HP) and heparan sulfate (HS), including residues 23–52, 53–93, and 110–128 (Pan et al., 2002; Warner et al., 2002). GAGs also regulate the cellular localization of PrP^*C*^ (Shyng et al., 1995; Taylor et al., 2009) and inhibit formation of PrP^*Sc*^ in cells and in animal models (Caughey and Raymond, 1993; Doh-ura et al., 2004). Despite the importance of GAGs in prion biology, the specific glycan structures that interact with different regions of PrP, and the kinetics of these interactions, have not been systematically investigated. This information is important for understanding the normal function of PrP^*C*^, its transformation into PrP^*Sc*^, and how the latter process could be inhibited for therapeutic effect.

**FIGURE 2:**
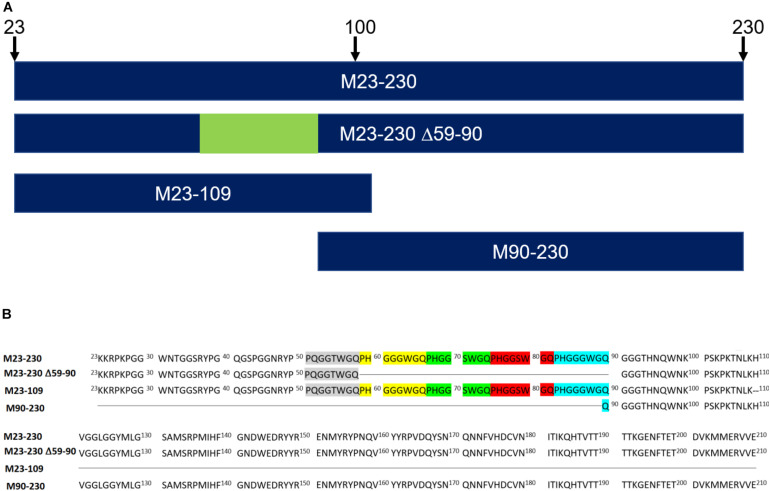
Recombinant murine PrP constructs. **(A)** Visual representation of primary sequences. **(B)** Recombinant murine PrP constructs lack signal peptide (1–22) and GPI anchor (231–254). The five octapeptide repeats are highlighted in gray, yellow, green, red, and turquois. M23–230 ΔM90–230 is a deletion mutant. M23–109 is the N-terminal domain whereas M90–230 is the C-terminal domain.

In this study, we employed direct binding surface plasmon resonance (SPR) assay to characterize the kinetics of heparin binding to four recombinant murine PrP constructs (Figures 2A,B): (1) full length (M23–230), (2) a deletion mutant lacking the four histidine-containing octapeptide repeats (M23–230 Δ59–90), (3) the isolated N-terminal domain (M23–109), and (4) the isolated C-terminal domain (M90–230). Additionally, we identified the specific structural determinants required for GAG binding to the four PrP constructs using an SPR competition assay with chemically defined derivatives of heparin and other GAGs.

## Materials and Methods

### Recombinant PrP

Four different mouse PrP (moPrP) constructs (Figure 2) were prepared by Prof. David Harris’s Group (Boston University). These included 1 mg of full length construct: M23–230 (*MW* = 23,061 Da); 1 mg of the N-terminal domain: M23–109 (*MW* = 9,142 Da); 0.5 mg of delta OR: M23–230 Δ59–90 (*MW* = 19,763 Da); and 2.5 mg of the C-terminal domain: M90–230 (*MW* = 16,013 Da). These proteins were prepared in *E. coli*, and purified as previously described, and characterized by SDS-PAGE and circular dichroism (CD) (Wu et al., 2017; McDonald et al., 2019).

### Glycosaminoglycans

The GAGs used were porcine intestinal heparin (16 kDa), low molecular weight heparin (LMWH) (5 kDa, enoxaparin, Sanofi-Aventis) and porcine intestinal heparan sulfate (12 kDa, Celsus Laboratories, Cincinnati, OH); chondroitin sulfate A (CSA, 20 kDa) from porcine rib cartilage (Sigma, St. Louis, MO), dermatan sulfate (also known as chondroitin sulfate B, CSB, 30 kDa, from porcine intestine, Sigma), dermatan disulfate (4-,6-disulfo DS, 33 kDa, Celsus) prepared through the chemical 6-*O*-sulfonation of dermatan sulfate (Van Gorp et al., 1999), chondroitin sulfate C (CSC, 20 kDa, from shark cartilage, Sigma), chondroitin sulfate D (CSD, 20 kDa, from whale cartilage, Seikagaku, Tokyo, Japan), chondroitin sulfate E (CSE, 20 kDa from squid cartilage, Seikagaku) and keratan sulfate (KS, 14.3 kDa) was isolated from bovine cornea in Linhardt Lab (Weyers et al., 2013). *N*-desulfated heparin (14 kDa) and 2-*O*-desulfated IdoA heparin (13 kDa) were all prepared based on Yates et al. (1996). 6-*O*-desulfated heparin (13 kDa) was kindly provided by Prof. Lianchun Wang from University of South Florida. Heparin oligosaccharides included tetrasaccharide (dp4), hexasaccharide (dp6), octasaccharide (dp8), decasaccharide (dp10), dodecasaccharide (dp12), tetradecasaccharide (dp14), hexadecasaccharide (dp16), and octadecasaccharide (dp18) and were prepared from controlled partial heparin lyase 1 treatment of bovine lung heparin (Sigma) followed by size fractionation. The chemical structures of these GAGs are shown in Figure 1.

### Preparation of Heparin Biochip

BIAcore 3000 SPR instrument and sensor SA chips were from GE Healthcare (Uppsala, Sweden). SPR measurements were performed on a BIAcore 3000 operated using BIAcore 3000 control and BIAevaluation software (version 4.0.1). Heparin was biotinylated by our previous protocol with minor modification (Kim et al., 2018). Heparin (2 mg) and 2 mg of amine–PEG_3_–Biotin (Thermo Fisher Scientific, Waltham, MA) were mixed with 10 mg of NaCNBH_3_ in 200 μL of H_2_O for the initial reaction, which was performed at 70°C for 24 h, and then a further 10 mg of NaCNBH_3_ was added to continue the reaction for another 24 h. Upon completion of this reaction, the mixture was desalted with a spin column (3000 molecular weight cut-off). The biotinylated heparin was immobilized to streptavidin (SA) chip based on the manufacturer’s protocol. In brief, 20 μL solution of the heparin-biotin conjugate (0.1 mg/mL) in HBS-EP running buffer was injected over flow cell 2 (FC2) of the SA chip at a flow rate of 10 μL/min. The successful immobilization of heparin was confirmed by the observation of a ∼200 resonance unit (RU) increase in the sensor chip. The control flow cell (FC1) was prepared by 1 min injection with saturated biotin.

### Measurement of Interaction Between Heparin and Prp Using Biacore

The PrP samples were diluted in HBS-EP buffer (0.01 M HEPES, 0.15 M NaCl, 3 mM EDTA, 0.005% surfactant P20, pH 7.4). Different dilutions of PrP samples were injected at a flow rate of 30 μL/min. At the end of the sample injection, the same buffer was flowed over the sensor surface to facilitate dissociation. After a 3 min dissociation time, the sensor surface was regenerated by injecting with 30 μL of 2 M NaCl to get fully regenerated surface. The response was monitored as a function of time (sensorgram) at 25°C.

### Solution Competition Study Between Heparin on Chip Surface and Heparin-Derived Oligosaccharides in Solution Using SPR

PrP (63 or 125 nM) mixed with 1,000 nM of heparin oligossacharides, including dp4, dp6, dp8, dp10, dp12, dp14, dp16, and dp18 in HBS-EP buffer were injected over heparin chip at a flow rate of 30 μL/min, respectively. After each run, the dissociation and the regeneration were performed as described above. For each set of competition experiments on SPR, a control experiment (only protein without any heparin or oligosaccharides) was performed to make sure the surface was completely regenerated and that the results obtained between runs were comparable. Statistical analysis was conducted using a student’s *t*-test.

### Solution Competition Study Between Heparin on Chip Surface and GAGs, Chemical Modified Heparin in Solution Using SPR

For testing of inhibition by other GAGs and chemical modified heparins of the PrP-heparin interaction, PrP at 63 or 125 nM was pre-mixed with 1,000 nM of GAG or chemical modified heparin and injected over the heparin chip at a flow-rate of 30 μL/min. After each run, a dissociation period and regeneration protocol was performed as described above. Statistical analysis was conducted using a student’s *t*-test.

## Results

### Kinetics Measurements of Prp-Heparin Interactions

**FIGURE 3:**
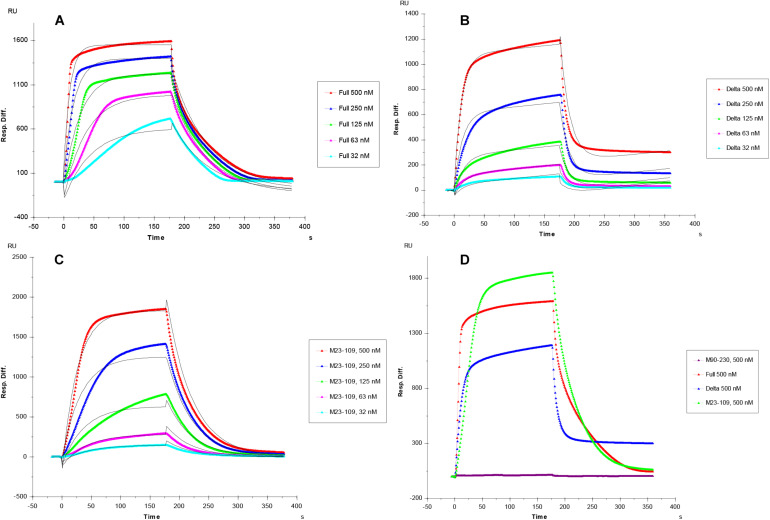
**(A–C)** SPR sensorgrams of PrP-heparin interaction for kinetic measurements. **(A)** Full length PrP (M23–230). **(B)** Delta PrP (M23–230 Δ59–90). **(C)** N-terminal PrP (M23–109), concentrations of PrP protein injected (from top to bottom): 500, 250, 125, 63, and 32 nM, respectively. The black curves are the fitting curves using models from BIAevaluate 4.0.1. **(D)** SPR sensorgrams comparison (500 nM injection) of Full length PrP, Delta PrP, M23–109 PrP, and M90–230 PrP-heparin interaction. All measurements **(A–D)** were made using the same SPR chip immobilized with heparin (average molecular weight ∼15 kDa).

Kinetic curves calculated from sensorgrams fitted to a 1:1 Langmuir model from BIAevaluate 4.0.1 demonstrate binding affinity, K_*D*_, values in the following order: full length PrP (1.1 × 10^–7^ M), M23–109 PrP (K_*D*_ = 7.1 × 10^–7^ M), and M23–230 Δ59–90 PrP (3.3 × 10^–6^ M) shown in Table 1 and Figures 3A–C. M90–230 PrP showed negligible binding to heparin even at the highest concentration used in this direct binding assay for all samples, 500 nM (Figure 3D). Similarly, association rate constant (k_*a*_) was greatest for full length PrP (1.6 × 10^5^) followed by M23–109 PrP (3.2 × 10^4^) and M23–230 Δ59–90 PrP (2.1 × 10^4^) (Table 1 and Figures 3A–C). Of the previously identified, putative heparin binding motifs (23–52, 53–93, and 110–128) (Pan et al., 2002; Warner et al., 2002), 23–52 and 53–93 appear to contribute to binding affinity most significantly. The smaller and flexible conformation of M23–109 PrP may additionally facilitate tighter binding to heparin. For example, M23–109 PrP may be able to bind sub-populations of immobilized heparin (i.e., shorter chain length heparin) that full length PrP does not.

**TABLE 1:** Summary of kinetic data of PrP protein- heparin interactions*.

Interaction	k_*a*_ *(1/MS)*	k_*d*_ *(1/S)*	*K_*D*_ (M)*
PrP Full length/Heparin	1.6 × 10^5^ (±4.1 × 10^3^)	0.017 (±3.0 × 10^–4^)	1.1 × 10^–7^
PrP M23–230 Δ59–90/Heparin	2.1 × 10^4^ (±1.5 × 10^3^)	0.069 (±9.9 × 10^–4^)	3.3 × 10^–6^
PrP M23–109/Heparin	3.2 × 10^4^ (±996)	0.023 (±2.3 × 10^–4^)	7.1 × 10^–7^

*The data with (±) in parentheses are the standard deviations (SD) from global fitting of five injections.

### Solution Competition Study on the Interactions Between the Immobilized Heparin With Prp Constructs to Heparin-Derived Oligosaccharides Using SPR

Solution/surface competition experiments were performed by SPR to examine the effect of the chain length of heparin on the heparin-PrP interactions. Different chain length heparin-derived oligosaccharides (from dp4 to dp18) at 1,000 nM were used in the competition study. LMWH (∼5 kDa) and unfractionated heparin (12–15 kDa) at 1,000 nM were also tested for their ability to inhibit PrP-heparin interactions.

**FIGURE 4:**
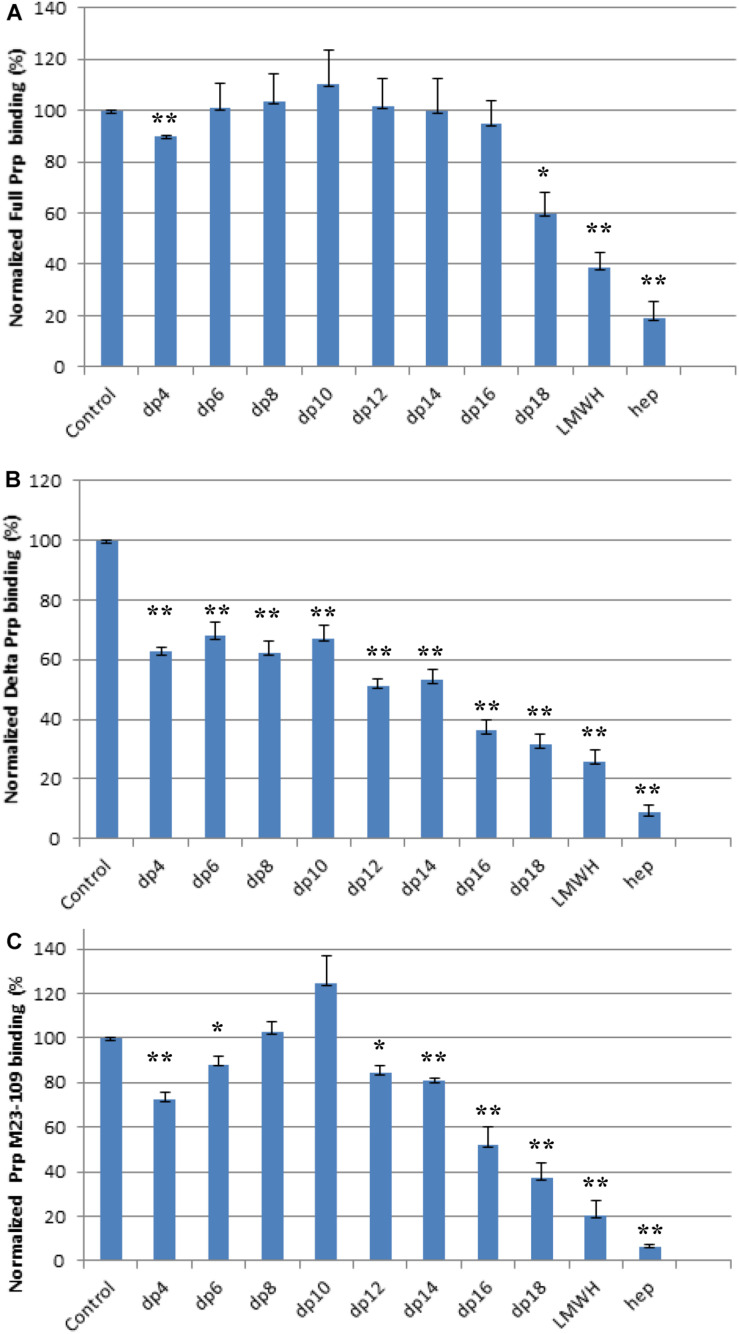
Bar graphs (based on triplicate experiments with standard deviation) of normalized PrP binding preference to surface heparin by competing with different size of heparin oligosaccharides in solution. One asterisk and two asterisks denote the statistical comparison between control and each sample (**p < 0.01; *p < 0.05). **(A)** Full length PrP (M23–230), concentration was 63 nM, concentrations of heparin oligosaccharides in solution were 1,000 nM. **(B)** Delta PrP (M23–230 Δ59–90) concentration was 125 nM, concentrations of heparin oligosaccharides in solution were 1,000 nM. **(C)** N-terminal PrP (M23–109) concentration was 125 nM, concentrations of heparin oligosaccharides in solution were 1,000 nM. All measurements **(A–C)** were made using the same SPR chip immobilized with heparin (average molecular weight ∼15 kDa).

For the full length PrP, inhibition effects of heparin oligosaccharides, LMWH, and unfractionated heparin were chain-length-dependent (Figure 4A). Negligible competition was observed when 1,000 nM of oligosaccharides (dp 4 to dp 16) present in the full length PrP protein solution (Figure 4A). The longer chain length heparin oligosaccharide, dp18, however, inhibited the binding of full length PrP to the surface heparin by 40% (Figure 4A). LMWH and unfractionated heparin inhibited PrP-heparin interactions more effectively, by 60 and 80%, respectively (Figure 4A). These results demonstrate that full length PrP prefers bindings to longer heparin chains.

Similarly, longer chain length heparin and heparin oligosaccharides inhibited M23–230 Δ59–90 PrP and heparin interactions more effectively (Figure 4B). However, the percent inhibition was greater for all compounds tested. Dp4-dp14 provided ∼40% inhibition to M23–230 Δ59–90 PrP (Figure 4B), which could be reached starting at dp18 for the full length PrP (Figure 4A). Unfractionated heparin inhibited M23–230 Δ59–90 PrP and heparin interactions by 90% (Figure 4B). M23–230 Δ59–90 PrP has the same primary amino acid sequence as full length PrP except for deletion of majority of the octapeptide repeats (59–90) and lacks one putative heparin binding motif at residues 53–93 (Figure 2). This lack of this binding motif and the potential alteration on the three-dimensional structure/conformation in the absence of residues 59–90 may have weakened the interactions between delta PrP and immobilized heparin surface allowing greater inhibition by heparin and heparin oligosaccharides at same concentration.

The N-terminal domain, M23–109 PrP demonstrated different mode of inhibition by heparin and heparin oligosaccharides (Figure 4C). dp4 inhibits M23–109 PrP and heparin interactions by ∼20%. However, this inhibition decreases with chain length up to dp 10, with the latter actually causing increased binding to surface heparin. From dp 12 to unfractionated heparin, the inhibition increases in a chain-length-dependent fashion. The N-terminal domain has two putative heparin binding motifs in 23–52 and 53–93 (Pan et al., 2002; Warner et al., 2002) and the 3-D conformation/folding may be altered from that of the full length PrP allowing heparin binding differently. For example, some of these regions may be exposed to the surface to more readily interact with shorter length heparin oligosaccharide, dp4. There is evidence that the N-terminal domain of PrP^*C*^ physically interacts with the C-terminal domain (McDonald et al., 2019) and the absence of this interaction in M23–109 might also influence the heparin binding characteristics of the latter protein. Finally, it is possible that shorter oligosaccharides (up to dp10) actually stabilize the structure of the N-terminal domain in such a way as to increase binding to surface heparin in the SPR experiments.

### SPR Solution Competition Study of Various GAGs

**FIGURE 5:**
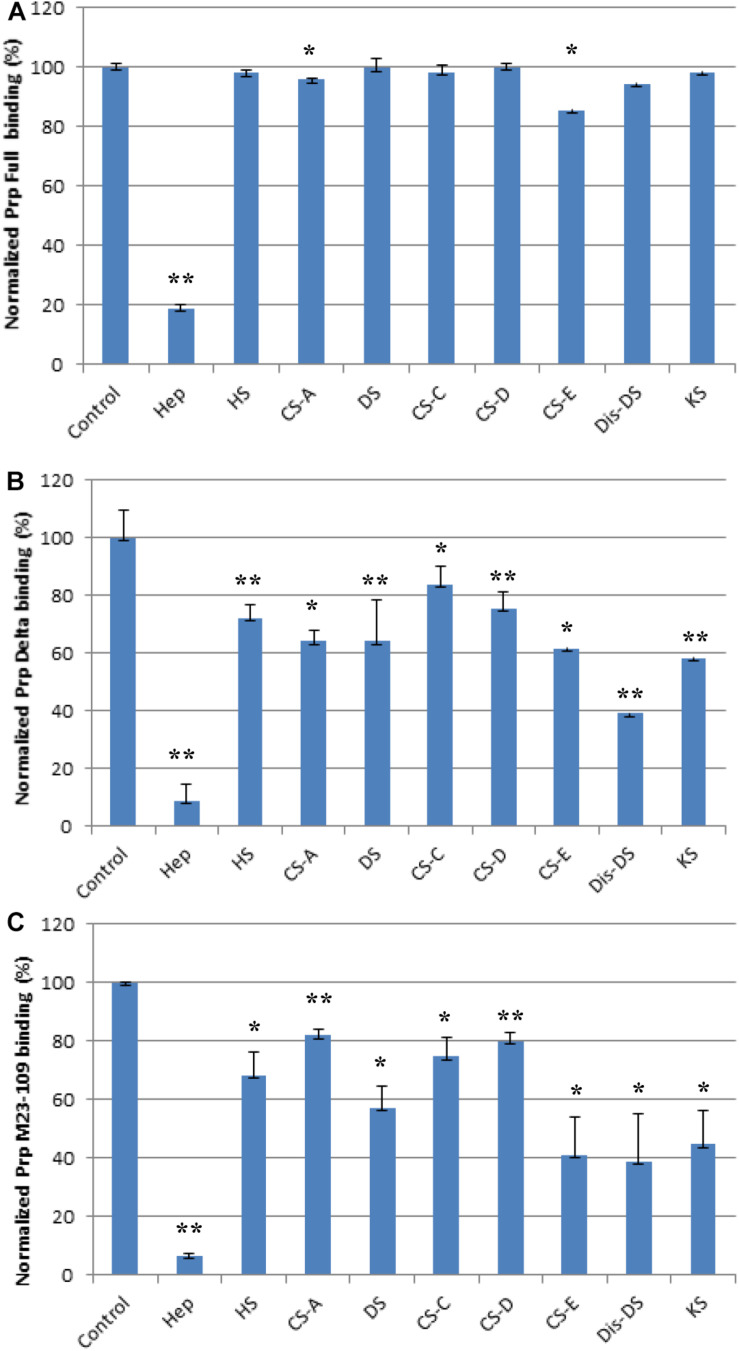
Bar graphs (based on triplicate experiments with standard deviation) of normalized PrP binding preference to surface heparin by competing with different GAGs. One asterisk and two asterisks denote the statistical comparison between control and each sample (**p < 0.01; *p < 0.05). **(A)** Full length PrP (M23–230) concentration was 63 nM, concentrations of GAGs in solution were 1,000 nM. **(B)** Delta PrP (M23–230 Δ59–90) concentration was 125 nM, concentrations of GAGs in solution were 1,000 nM. **(C)** N-terminal PrP (M23–109) concentration was 125 nM, concentrations of GAGs in solution were 1,000 nM. All measurements **(A–C)** were made using the same SPR chip immobilized with heparin (average molecular weight ∼15 kDa).

We screened inhibition capability of GAGs of different structures (Figure 1), including unfractionated heparin, HS, chondroitin sulfate type A (CS-A), CS-C, CS-D, CS-E, DS, disulfated DS (Dis-DS), and keratan sulfate (KS), against interactions between PrP constructs and immobilized heparin (Figure 5). All GAGs tested were used at 1,000 nM. For full length PrP, only unfractionated heparin was capable of inhibiting PrP-heparin interactions by 80% while the rest of GAGs showed negligible inhibition (Figure 5A). Unfractionated heparin inhibited M23–230 Δ59–90 PrP and heparin interactions by ∼90% and varying degree of inhibition was observed by other GAGs ranging from 20 to 60% inhibition (Figure 5B). This reinforces the idea of weakened binding interaction to immobilized heparin due to lack of one putative heparin binding motif and potential change in 3-D structure as described above. Lastly, inhibition ranging from 20 to 90% was demonstrated by various GAGs for inhibiting M23–109 PrP-heparin binding (Figure 5C), however, the preferred structure of GAG was different from those of full length or M23–230 Δ59–90 PrP, suggesting a different mode of binding then was observed in competition assays utilizing varying chain length heparin oligosaccharides (Figure 4C).

### SPR Solution Competition Study of Chemically Modified Heparin Derivatives

**FIGURE 6:**
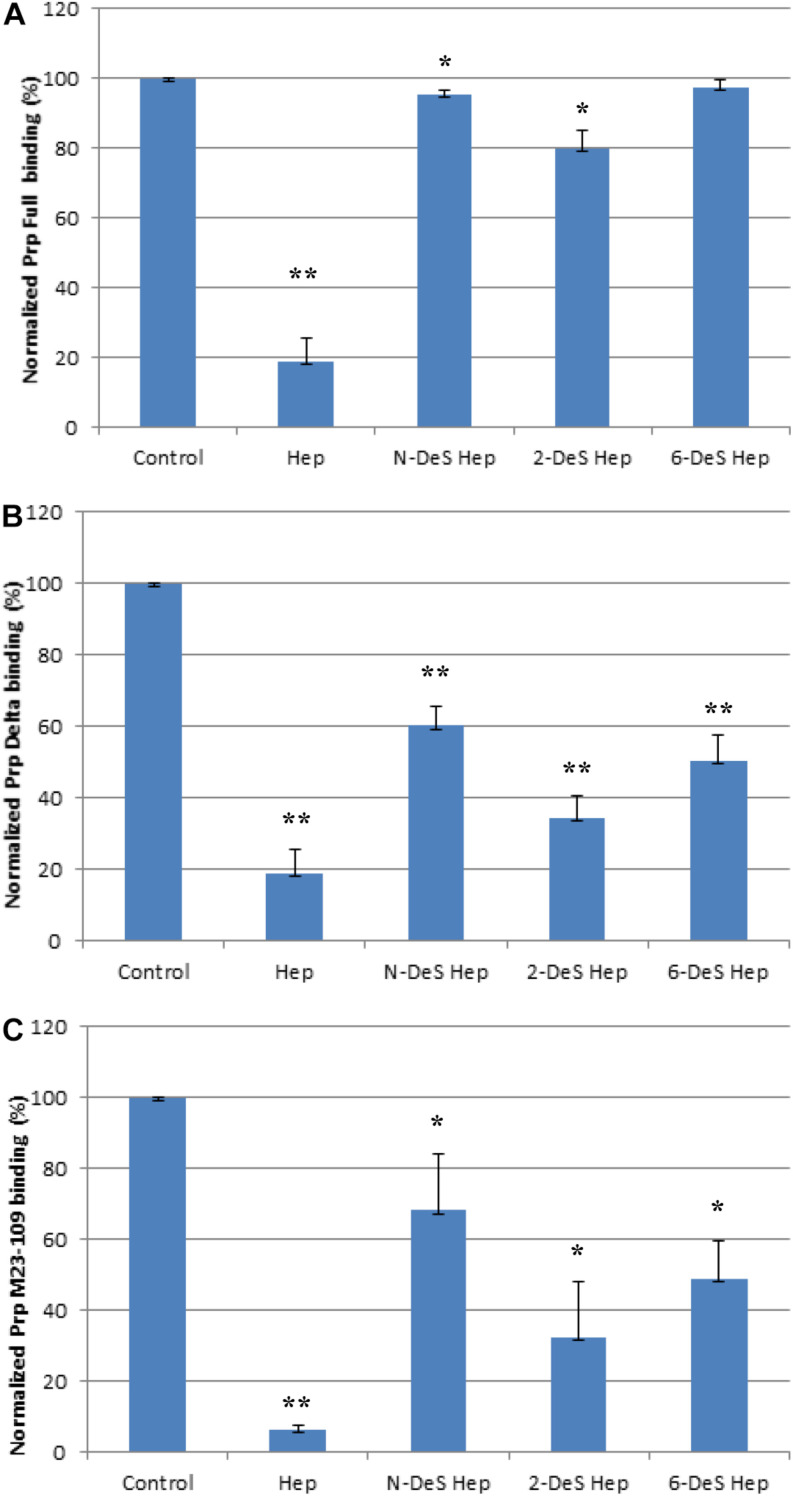
Bar graphs (based on triplicate experiments with standard deviation) of normalized Prp (Full) binding preference to surface heparin by competing with different chemical modified heparins in solution. One asterisk and two asterisks denote the statistical comparison between control and each sample (**p < 0.01; *p < 0.05). **(A)** Full length PrP (M23–230) concentration was 63 nM, concentrations of modified heparins in solution were 1,000 nM. **(B)** Delta PrP (M23–230 Δ59–90) concentration was 125 nM, concentrations of modified heparins in solution were 1,000 nM. **(C)** N-terminal PrP (M23–109) concentration was 125 nM, concentrations of modified heparins in solution were 1,000 nM. All measurements **(A–C)** were made using the same SPR chip immobilized with heparin (average molecular weight ∼15 kDa).

Next, we determined if *N*-, *2-O*, *3-O*, and *6-O*-sulfation on heparin were required for efficient binding to PrP constructs using chemically modified heparin derivatives. Of these heparin derivatives, only 2-DeS hep inhibited full length PrP and heparin interactions by 20% (Figure 6A). *N*- and *6-O* desulfated heparin derivatives, however, did not inhibit PrP and heparin interactions (Figure 6A). Unfractionated heparin has an additional *3-O* sulfation, which may be responsible for forming electrostatic interactions with surface accessible basic residues on the putative heparin binding motifs on the full length PrP. For both M23–230 Δ59–90 PrP and M23–109 PrP, however, all of the heparin derivatives inhibited PrP and heparin interactions (Figures 6B,C). These findings further suggest the importance of presence of all three putative heparin binding motifs, which also allow native conformation of full length PrP, for efficient binding to heparin.

## Discussion

Our investigation shows that full length PrP (M23–230) binds heparin with greatest binding affinity (K_*D*_ = 0.11 μM) followed by the N-terminus region PrP (M23–109) (K_*D*_ = 0.71 μM), and mutant PrP (M23–230 Δ59–90) (K_*D*_ = 3.3 μM) (Figure 3 and Table 1). The C-terminus region PrP (M90–230) exhibited negligible binding. Comparable binding affinities between full length PrP and M23–109 PrP confirm that the major heparin binding sites are localized within the N-terminal region (23–52) and the octapeptide repeats (53–90), but not in the C-terminal region (Pan et al., 2002; Warner et al., 2002). The polybasic amino acid segment (residues 23–31) within the putative heparin binding motif (23–52) modulates ion channel activity of PrP, perhaps modulated by GAG binding (Le et al., 2019). The region containing the four histidine-containing octapeptide repeats [PHGG(G/S)WGQ] (53–93) was previously determined to possess an additional putative heparin binding motif (Pan et al., 2002; Warner et al., 2002). Lacking this region reduced heparin binding ability (Table 1 and Figures 3–6). Copper ions binding to this region on the PrP^*C*^ on the neuronal cell surface results in rapid clathrin-dependent endocytosis of PrP^*C*^ (Hooper et al., 2008). heparin binding to bovine PrP^*C*^ is copper dependent (Andrievskaia et al., 2007). Copper and other metal ions interact with heparin or heparan sulfate to modulate heparin binding to proteins (Zhang et al., 2014; Kim et al., 2018). Binding of sulfated GAGs and copper ions to the N-terminal domain regulates ion channel activity and other toxic effects of PrP^*C*^ (Wu et al., 2017; Le et al., 2019).

In the competition SPR binding assays, we determined structural preferences of PrP binding. Both full length PrP and M23–230 Δ59–90 PrP show similar trend of preferred binding to heparin oligosaccharides with longer chain length (Figures 4A,B). By lacking four of the five octapeptide repeats, the M23–230 Δ59–90 PrP shows lower binding affinity to immobilized heparin allowing heparin oligosaccharides to inhibit this interaction by a greater extent. The isolated N-terminal domain (M23–109), however, showed a different mode of competition with heparin, where both shorter (dp4 and dp6) or longer (dp12-unfractionated heparin) chain length heparin than dp8 and dp10 exhibited greater level of inhibition (Figure 4C). Lacking a heparin binding motif at 110–128 as well as intramolecular interactions with the C-terminal domain appears to have altered the original mode of heparin binding. While these are interesting trends, it is also possible that the varying levels of magnitude may be due to differences in the binding affinities against heparin.

The competition assay results of screening various types of GAGs further demonstrate that the positions of basic residues in PrP are important for GAG binding, likely by determining the spatial arrangement of electrostatic interactions with carboxylate and sulfate groups on the GAG molecules. The last set of competition assays using chemically modified heparin derivatives has suggested that 3-O sulfation is most important for heparin binding to full length PrP, whereas *N*-, 2-*O*, 3-*O*, and 6-*O*-sulfation appears to be important for heparin interactions with M23–230 Δ59–90 PrP or M23–109 PrP (Figures 6B,C). Overall 3-D structural changes in PrP lacking heparin-binding motifs (residues 53–90 or 110–128) alter types of GAGs and sulfation patterns of heparin it preferentially binds to; and this should be considered in developing designer GAGs as PrP therapeutic. Similarly, based on the results from the competition assay using heparin oligosaccharides (Figure 4), we conjecture that the varying levels of inhibition may be the result of varying heparin-binding strength for three PrP constructs. In human PrP^*C*^, 2-*O*-sulfate groups, but not 6-*O*-sulfate position, are required for heparin recognition (Warner et al., 2002).

In summary, we have characterized binding interactions between four different PrP constructs [full length (M23–230), M23–230 Δ59–90, N- and C-terminal domains] and different forms of GAGs varying in their structures. By SPR direct binding assays, we determined the kinetics of these PrP-heparin interactions, and confirmed that previously identified, putative heparin binding motifs were essential for the binding. Competition assays utilizing varying chain length of heparin and heparin oligosaccharides revealed that full length and M23–230 Δ59–90 PrP prefer binding longer chain length heparin, while the N-terminal domain of PrP had a different mode of binding. Binding of full length PrP to heparin was effectively inhibited only by unfractionated heparin. However, M23–230 Δ59–90 and the N-terminal domain exhibited preferential binding to various types of GAGs, with Dis-DS being the best inhibitor for both (besides heparin). Screening of chemically modified heparin derivatives in PrP-heparin competition assays demonstrated that *3-O* sulfation is critical for full length PrP and heparin binding while M23–230 Δ59–90 and *N*-terminal domain require all sulfation positions. Our findings on the structural requirements for efficient binding to these PrP constructs lays the foundation for designing tailored GAG inhibitors targeting different regions within the PrP molecule. Such inhibitors may be useful for controlling the biological and pathophysiological activities of PrP.

## Data Availability Statement

The original contributions presented in the study are included in the article/supplementary material, further inquiries can be directed to the corresponding author/s.

## Author Contributions

SK and FZ performed SPR assays, analyzed the data, wrote, and revised the manuscript. RL conceived, designed the project, and revised the manuscript. DH prepared the PrP proteins and revised the manuscript. All authors contributed to the article and approved the submitted version.

## Conflict of Interest

The authors declare that the research was conducted in the absence of any commercial or financial relationships that could be construed as a potential conflict of interest.

## References

[B1] Andrievskaia O. Potetinova Z. Balachandran A. Nielsen K. (2007). Binding of bovine prion protein to heparin: a fluorescence polarization study. *Arch. Biochem. Biophys.* 460 10–16. 10.1016/j.abb.2007.02.001 17353004

[B2] Caughey B. Raymond G. J. (1993). Sulfated polyanion inhibition of scrapie-associated PrP accumulation in cultured cells. *J. Virol.* 67 643–650. 10.1128/jvi.67.2.643-650.1993 7678300 PMC237415

[B3] Doh-ura K. Ishikawa K. Murakami-Kubo I. Sasaki K. Mohri S. Race R. (2004). Treatment of transmissible spongiform encephalopathy by intraventricular drug infusion in animal models. *J. Virol.* 78 4999–5006. 10.1128/jvi.78.10.4999-5006.2004 15113880 PMC400350

[B4] Hooper N. M. Taylor D. R. Watt N. T. (2008). Mechanism of the metal-mediated endocytosis of the prion protein. *Biochem. Soc. Trans.* 36 1272–1276. 10.1042/BST0361272 19021539

[B5] Kim S. Y. Jin W. Sood A. Montgomery D. W. Grant O. C. Fuster M. M. (2020). Characterization of heparin and severe acute respiratory syndrome-related coronavirus 2 (SARS-CoV-2) spike glycoprotein binding interactions. *Antiviral Res.* 181:104873. 10.1016/j.antiviral.2020.104873 32653452 PMC7347485

[B6] Kim S. Y. Zhang F. Gong W. Chen K. Xia K. Liu F. (2018). Copper regulates the interactions of antimicrobial piscidin peptides from fish mast cells with formyl peptide receptors and heparin. *J. Biol. Chem.* 293 15381–15396. 10.1074/jbc.RA118.001904 30158246 PMC6177607

[B7] Le N. T. T. Wu B. Harris D. A. (2019). Prion neurotoxicity. *Brain Pathol.* 29 263–277. 10.1111/bpa.12694 30588688 PMC6894960

[B8] Linhardt R. J. Toida T. (2004). Role of glycosaminoglycans in cellular communication. *Acc. Chem. Res.* 37 431–438. 10.1021/ar030138x 15260505

[B9] McDonald A. J. Leon D. R. Markham K. A. Wu B. Heckendorf C. F. Schilling K. (2019). Altered domain structure of the prion protein caused by Cu2(binding and functionally relevant mutations: analysis by cross-linking, MS/MS, and NMR. *Structure* 27 907–922.e5. 10.1016/j.str.2019.03.008 30956132 PMC6736647

[B10] Mercer R. C. C. Mcdonald A. J. Bove-fenderson E. Fang C. Wu B. Harris D. A. (2018). “Prion diseases,” in *The Molecular and Cellular Basis of Neurodegenerative Diseases: Underlying Mechanisms*, 1st Edn., ed. M. Wolfe (London: Academic Press), 23–56. Available online at: https://www.elsevier.com/books/the-molecular-and-cellular-basis-of-neurodegenerative-diseases/wolfe/978-0-12-811304-2

[B11] Pan T. Wong B. S. Liu T. Li R. Petersen R. B. Sy M. S. (2002). Cell-surface prion protein interacts with glycosaminoglycans. *Biochem. J.* 368 81–90. 10.1042/BJ20020773 12186633 PMC1222984

[B12] Shyng S. L. Lehmann S. Moulder K. L. Harris D. A. (1995). Sulfated glycans stimulate endocytosis of the cellular isoform of the prion protein, PrPC, in cultured cells. *J. Biol. Chem.* 270 30221–30229. 10.1074/jbc.270.50.30221 8530433

[B13] Solomon I. H. Khatri N. Biasini E. Massignan T. Huettner J. E. Harris D. A. (2011). An N-terminal polybasic domain and cell surface localization are required for mutant prion protein toxicity. *J. Biol. Chem.* 286 14724–14736. 10.1074/jbc.M110.214973 21385869 PMC3077669

[B14] Taylor D. R. Whitehouse I. J. Hooper N. M. (2009). Glypican-1 mediates both prion protein lipid raft association and disease isoform formation. *PLoS Pathog.* 5:1000666. 10.1371/journal.ppat.1000666 19936054 PMC2773931

[B15] Van Gorp C. L. Brister S. J. Buchanan M. R. Linhardt R. J. (1999). *Dermatan Disulfate, An Inhibitor of Thrombin Generation and Activation.* Patent No: US5922690A. Available online at: https://patents.google.com/patent/US5922690A/en

[B16] Vieira T. C. R. G. Reynaldo D. P. Gomes M. P. B. Almeida M. S. Cordeiro Y. Silva J. L. (2011). Heparin binding by murine recombinant prion protein leads to transient aggregation and formation of rna-resistant species. *J. Am. Chem. Soc.* 133 334–344. 10.1021/ja106725p 21142149

[B17] Warner R. G. Hundt C. Weiss S. Turnbull J. E. (2002). Identification of the heparan sulfate binding sites in the cellular prion protein. *J. Biol. Chem.* 277 18421–18430. 10.1074/jbc.M110406200 11882649

[B18] Westergard L. Turnbaugh J. A. Harris D. A. (2011). A nine amino acid domain is essential for mutant prion protein toxicity. *J. Neurosci.* 31 14005–14017. 10.1523/JNEUROSCI.1243-11.2011 21957261 PMC3227396

[B19] Weyers A. Yang B. Solakyildirim K. Yee V. Li L. Zhang F. (2013). Isolation of bovine corneal keratan sulfate and its growth factor and morphogen binding. *FEBS J.* 280 2285–2293. 10.1016/j.pestbp.2011.02.012.Investigations 23402351 PMC3651742

[B20] Wu B. McDonald A. J. Markham K. Rich C. B. McHugh K. P. Tatzelt J. (2017). The N-terminus of the prion protein is a toxic effector regulated by the C-terminus. *eLife* 6:e23473. 10.7554/eLife.23473 28527237 PMC5469617

[B21] Yates E. A. Santini F. Guerrini M. Naggi A. Torri G. Casu B. (1996). 1H and 13C NMR spectral assignments of the major sequences of twelve systematically modified heparin derivatives. *Carbohydr. Res.* 294 15–27. 10.1016/s0008-6215(96)90611-4 8962483

[B22] Zahn R. Liu A. Lührs T. Riek R. Von Schroetter C. Garcia F. L. (2000). NMR solution structure of the human prion protein. *Proc. Natl. Acad. Sci. U.S.A.* 97 145–150. 10.1073/pnas.97.1.145 10618385 PMC26630

[B23] Zhang F. Liang X. Beaudet J. M. Lee Y. Linhardt R. J. (2014). The effects of metal ions on heparin/heparin sulfate-protein interactions. *J. Biomed. Technol. Res.* 1 1–14. 10.1016/j.physbeh.2017.03.040 28890953 PMC5589159

